# Hybrid Dielectric-loaded Nanoridge Plasmonic Waveguide for Low-Loss Light Transmission at the Subwavelength Scale

**DOI:** 10.1038/srep40479

**Published:** 2017-01-16

**Authors:** Bin Zhang, Yusheng Bian, Liqiang Ren, Feng Guo, Shi-Yang Tang, Zhangming Mao, Xiaomin Liu, Jinju Sun, Jianying Gong, Xiasheng Guo, Tony Jun Huang

**Affiliations:** 1 Department of Engineering Science and Mechanics, The Pennsylvania State University, University Park, PA 16802, USA; 2 Department of Fluid Machinery and Engineering, School of Energy and Power Engineering, Xi’an Jiaotong University, Xi’an 710049, P. R. China; 3 MOE Key Laboratory of Thermo-Fluid Science and Engineering, School of Energy and Power Engineering, Xi’an Jiaotong University, Xi’an 710049, P. R. China; 4 Key Laboratory of Modern Acoustics (MOE), Department of Physics, Nanjing University, Nanjing 210093, P.R. China; 5 Department of Mechanical Engineering and Materials Science, Duke University, Durham, NC 27708, USA

## Abstract

The emerging development of the hybrid plasmonic waveguide has recently received significant attention owing to its remarkable capability of enabling subwavelength field confinement and great transmission distance. Here we report a guiding approach that integrates hybrid plasmon polariton with dielectric-loaded plasmonic waveguiding. By introducing a deep-subwavelength dielectric ridge between a dielectric slab and a metallic substrate, a hybrid dielectric-loaded nanoridge plasmonic waveguide is formed. The waveguide features lower propagation loss than its conventional hybrid waveguiding counterpart, while maintaining strong optical confinement at telecommunication wavelengths. Through systematic structural parameter tuning, we realize an efficient balance between confinement and attenuation of the fundamental hybrid mode, and we demonstrate the tolerance of its properties despite fabrication imperfections. Furthermore, we show that the waveguide concept can be extended to other metal/dielectric composites as well, including metal-insulator-metal and insulator-metal-insulator configurations. Our hybrid dielectric-loaded nanoridge plasmonic platform may serve as a fundamental building block for various functional photonic components and be used in applications such as sensing, nanofocusing, and nanolasing.

Surface plasmon polariton (SPP) has been identified as a key enabling technology for highly integrated photonic components and circuits, due to its unique potential for manipulating the flow of light at scales much smaller than the diffraction limit^1^,^2^,^3^,^4^,^5^,^6^,^7^. Among a variety of waveguiding configurations, plasmonic structures that incorporate metallic features are ideal for light transmission at the sub-wavelength scale^8^. A number of SPP-based waveguiding schemes, such as metallic nanoparticles^9^, nanowires^10^,^11^,^12^,^13^,^14^, stripes^15^, wedges^16^,^17^, grooves^18^,^19^, slots^20^,^21^,^22^ and dielectric-loaded structures^23^,^24^,^25^,^26^,^27^,^28^, have been proposed and demonstrated in recent years. In contrast to offering significantly better optical confinement than conventional all-dielectric waveguiding counterparts, the guiding performances of SPP configurations are still severely restricted by the fundamental tradeoff between confinement and loss^29^, which greatly hinders their practical implementations.

Recently, a new class of hybridized plasmonic guiding structures combining dielectric waveguiding with surface plasmon polariton transport has been proposed^30^,^31^,^32^, and it offers a promising solution to the limitations of traditional SPP waveguides. With the incorporation of additional low-index dielectric layers between metal structures and high-index dielectric configurations, these hybrid plasmonic waveguides (HPWs) allow both enhanced field localization and reduced transmission loss, as compared to most previously reported plasmonic structures. Their guiding performances render themselves as ideal candidates for realizing high-performance photonic components^27^,^33^,^34^,^35^,^36^,^37^,^38^,^39^,^40^, and they also offer great potential for a number of intriguing applications^41^,^42^,^43^,^44^,^45^. In addition to the conventional hybridized waveguide configurations, extensive efforts have also been devoted to the exploration of modified hybrid structures^32^,^46^,^47^,^48^,^49^,^50^,^51^,^52^,^53^,^54^,^55^,^56^,^57^. Though some of these novel configurations exhibit improved optical performance as opposed to their conventional hybrid counterparts, most of them still suffer from the tradeoff between modal attenuation and field localization. Moreover, due to additional fabrication complexities, many of these modified waveguides face great challenges when leveraged for practical applications. Therefore, there is a need for a simple but feasible way to reduce the propagation loss of traditional hybrid waveguide while maintaining its tight-field localization property.

Here in this article, we propose a new type of HPW by combining dielectric-loaded waveguiding with a traditional hybrid structure, which we refer to as a hybrid dielectric-loaded nanoridge plasmonic waveguide (HDLNRPW). In contrast to the previous hybrid wedge/ridge structures that incorporate metallic nanostructures^52^,^53^, the hybrid waveguides presented here take full advantage of dielectric nanoridges, which are beneficial for reducing the propagation loss and maintaining the tight field confinement. Based on systematic numerical simulations, we will show in detail the capability of the hybrid dielectric-loaded nanoridge waveguide in balancing the tradeoff between confinement and loss, and we will reveal its tolerance against fabrication errors. Moreover, we will discuss the possibility of applying the waveguide concept to other metal/dielectric structures, which will lay the foundation for future designs and investigations.

## Results

**Figure 1:**
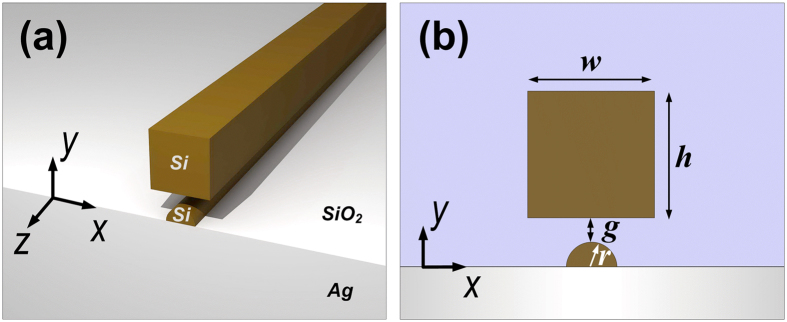
Schematics of the proposed HDLNRPW. (**a**) 3D layout of the hybrid waveguide. (**b**) Cross-section of the structure. The hybrid configuration consists of a silicon nanoridge-loaded semi-infinite silver substrate, which is separated from an upper silicon slab by the silica layer. The upper silicon slab has a width of *w* and a height of *h*, whereas the lower silicon nanoridge has a radius of *r*. The gap size, which is defined as the smallest distance between the lower and upper silicon nanostructures, is denoted by *g*. The silicon nanoridge is supposed to be at the center position (along *x* axis) with respect to the upper silicon slab unless stated otherwise.

Figure 1(a) and (b) show schematically three-dimensional (3D) and two-dimensional (2D) geometries of the studied hybrid waveguide, which consists of a silicon slab separated from a silver substrate by a thin silica layer, along with an additional silicon nanoridge sitting on top of the substrate. The unique hybrid gap region facilitates efficient light confinement and transport with moderate attenuation within the nanoscale low-index layers. To reveal the potential of the structure in offering both good confinement and low transmission loss, we used the finite element method (FEM)-based software COMSOL^TM^ to investigate its guiding properties at a telecommunication wavelength of 1550 nm. In our calculations, the refractive indices of SiO_2_, Si, and Ag were chosen to be n_l_ = 1.444, n_h_ = 3.476, and n_m_ = 0.1453 + 14.3587i^30^, respectively. Without loss of generality, semi-circular-shaped dielectric nanostructures were chosen as a proof-of-concept in the following studies. In our later discussions, we will show that our waveguide concept can also be applied to many other configurations with similar nanostructures, including those incorporating rectangular, semi-elliptical and triangular-shaped dielectric nanoridges.

**Figure 2:**
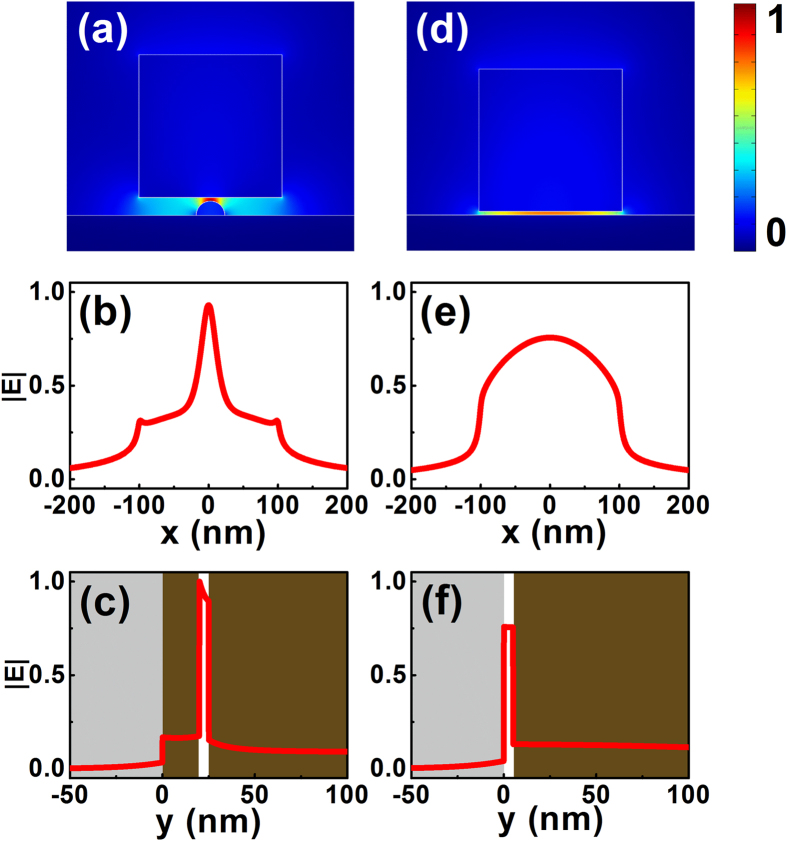
2D and 1D normalized electric field distributions for (**a**)–(**c**) HDLNRPW, and (**d**)–(**f**) traditional HPW. For both structures, the dimensions of the rectangular-shaped silicon slabs are fixed at *w* = *h* = 200 nm, while their gap sizes were both chosen as *g* = 5 nm. The radius of the silicon nanoridge was *r = *20 nm. The electric fields were normalized with respect to the power flow in each structure. The 1D field profiles show the normalized electric fields along the center of the gap region.

In Fig. 2, we show the normalized electric field distribution of the fundamental hybrid mode supported by a typical hybrid nanoridge plasmonic structure, and we compare this distribution with that of the conventional hybrid mode. In the calculations, both the HDLNRPW and the conventional HPW have the same gap size of 5 nm, and the dimensions of the silicon slabs for both structures are fixed at 200 nm × 200 nm. Due to the strong hybridization of the plasmonic and dielectric modes, significant field enhancement was observed inside the gap region for both cases. As illustrated from the 2D panel and 1D cross-sectional field plots, the local field enhancement of the proposed HDLNRPW is even more pronounced than the traditional hybrid structure in both horizontal and vertical directions, which can be attributed to the stronger effect induced by the lower silicon nanoridge. Our calculations also indicate that the HDLNRPW exhibits lower loss than the conventional hybrid waveguide, which is due to the larger distance between the upper silicon slab and the lower silver substrate. In the following section, we will illustrate the characteristics of the plasmonic mode guided by the proposed structure, and demonstrate the possibility of balancing the tradeoff between confinement and loss through tuning structural parameters of the waveguide.

**Figure 3:**
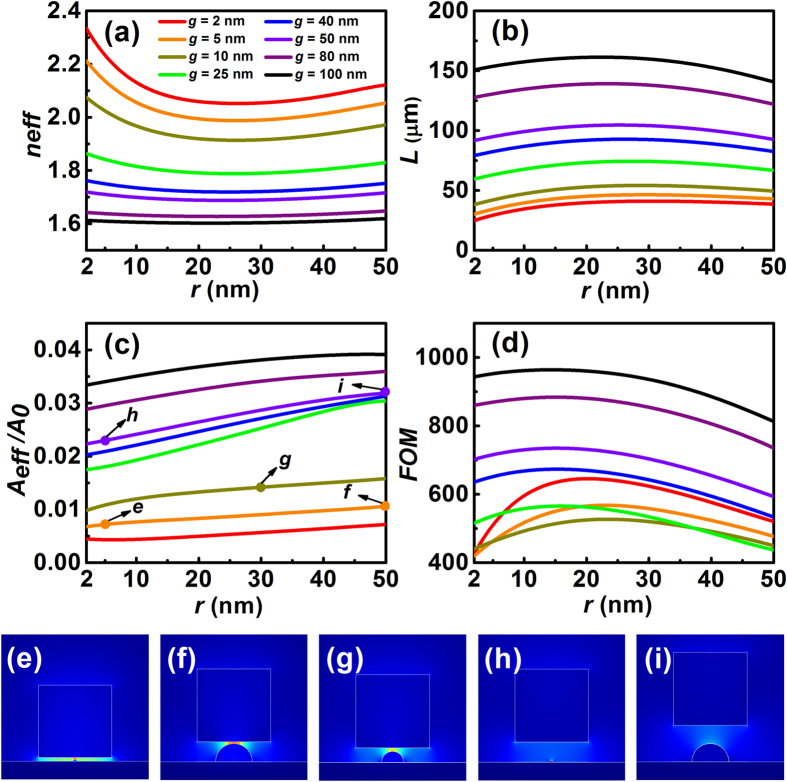
Modal properties and field distributions of HDLNRPWs with different *g* and *r*. (**a**)–(**d**) Dependence of modal characteristics on the radius of the nanoridge (*r*): (**a**) modal effective index (*n*_*eff*_); (**b**) propagation length (*L*); (**c**) normalized mode area (*A*_eff_*/A*_0_); (**d**) figure of merit (*FoM*). (**e**)–(**i**) Normalized electric field distributions for typical waveguides (corresponding to the configurations indicated in (**c**)): (**e**) *g* = 5 nm, *r* = 5 nm; (**f**) *g* = 5 nm, *r* = 50 nm; (**g**) *g* = 10 nm, *r* = 30 nm; (**h**) *g* = 50 nm, *r* = 5 nm; (**i**) *g* = 50 nm, *r* = 50 nm. All the fields are normalized with respect to the power flow in the cross-sections.

In order to reveal the unique potential of the proposed hybrid waveguide in providing tight optical confinement and great propagation length, we calculated the dependence of its modal properties on the gap size and the dimension of the silicon nanoridge. Firstly, we consider the following modal parameters, including the real part of the modal effective index (*n*_*eff*_ = Re(*N*_*eff*_)), the propagation length (*L*), the normalized mode area (*A*_*eff*_/*A*_*0*_), and the figure of merit (*FoM*) (see methods). As illustrated from the calculated results shown in Fig. 3(a) and (b), both the modal effective index and the propagation distance demonstrate non-monotonic behaviors with the variation of the silicon nanoridge. By contrast, the effective mode area increases slightly as the nanoridge becomes larger, and its value is much less than 1, indicating clearly the subwavelength confinement of the HDLNRPW. For waveguides with small nanoridges (e.g., *r* < 20 nm), the coupling between the silicon slab and the metal substrate was relatively strong, and it was further enhanced as the dimension of the nanoridge and/or the size of the gap decreased. This strengthened hybridization effect leads to an increased effective index, a shortened propagation distance, and a decreased mode area, as observed in Fig. 3(a)–(c). On the other hand, the overall features of the HDLNRPW were dominated by the silicon nanoridge when the dimension of the nanoridge reached a certain size (e.g., *r* > 30 nm). With the continuously enlarged nanoridgeas and enhanced modal effect index, a reduced propagation distance and an increased mode size were observed for the considered waveguides and the different gap sizes. Figure 3(b) illustrates that the largest propagation distances are typically obtained when the silicon nanoridge has a moderate radius (e.g., between 20–30 nm). This non-monotonic behavior of the propagation distance, together with the monotonic trend of the mode area, leads to the non-monotonic change of the *FoM*, as shown in Fig. 3(d). For the considered waveguiding structures, *FoM*s reach their maxima when the radius of the nanoridge was 15–25 nm. At these conditions, our proposed HDLNRPW features not only a much higher *FoM* but also a larger propagation distance as compared to its conventional hybrid waveguiding counterpart (see Supplementary Information for details). While compared to modified hybrid structures incorporating inverse metallic nanostructures^58^,^59^, our proposed waveguide enables much lower loss with subwavelengh field confinement. The propagation distance of HDLNRPW, ranging from tens to hundreds of microns, is more than one orders of magnitude greater than that reported in 58. These features indicate great potential of HDLNRPW for high-performance plasmon waveguiding at the sub-wavelength scale.

In addition to plotting the curves of different mode parameters, we also depict the electric field distributions for typical waveguide configurations, which are shown in Fig. 3(e)–(i). As illustrated from the field profiles, pronounced local field enhancement and tight optical confinement were achieved by waveguides with small gaps (Fig. 3(e)–(g)), due to the strong hybridization of the dielectric-loaded SPP and the dielectric mode supported by the silicon slab. By contrast, less notable field enhancements were achieved for waveguides with relatively large gap distances (Fig. 3(h)–(i)). Under these circumstances, the confinement of the hybrid waveguide is also weaker than the small-gap case, as indicated from the curves of the effective mode area shown in Fig. 3(c).

**Figure 4:**
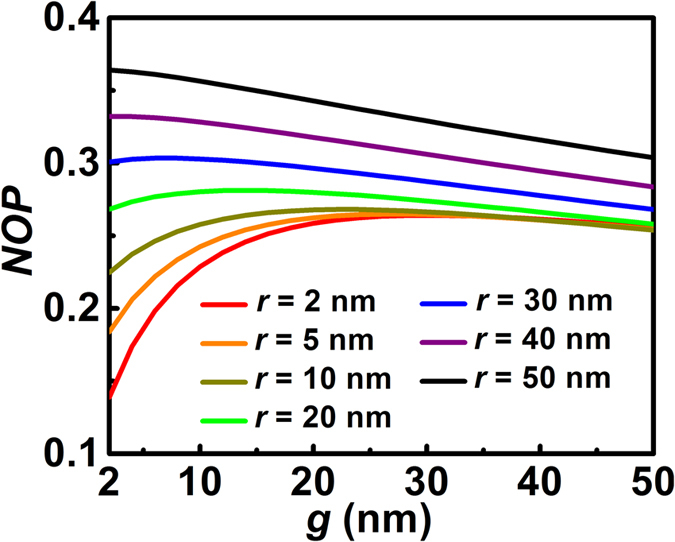
Normalized optical power (*NOP*) vs. gap size (*g*) for HDLNRPWs with different silicon nanoridges.

The field confinement of the HDLNRPW was further revealed by calculating the normalized optical power (*NOP*) inside the gap region (see methods). Here in Fig. 4 we show the dependence of *NOP* on the size of the gap for waveguides with different nanoridges. It is seen that the power ratio inside the gap exhibits a non-monotonic trend with the variation of *g* when *r* is relatively small (e.g., *r* < 40 nm), which indicates the existence of an optimal gap size for *NOP*. Such an optimal *g* shifts towards a smaller value when the size of the nanoridge increases. As illustrated in Fig. 4, the trend of *NOP* turns into a monotonic behavior when *r* becomes greater than 40 nm. Under these circumstances, the power confined inside the silicon nanoridge is significantly greater than the silica layer of the gap region. Further enlarging the gap size will lead to weakened field confinement inside the whole gap, as illustrated from the decreasing trends of the purple and black curves in Fig. 4. Our calculations show that through choosing appropriate *r* and *g*, the confinement of HDLNRPW can be significantly greater than that of the conventional hybrid waveguide inside the gap region. This tight field confinement, along with pronounced local field enhancement, small mode size, and large propagation distance, can potentially enable applications in active waveguides and nanolasers^33^,^36^,^40^,^60^, nonlinear devices^43^,^45^ as well as high-sensitivity optical sensors^61^,^62^,^63^,^64^,^65^,^66^,^67^. On the other hand, combining our HDLNRPW concept with novel materials such as graphene^68^,^69^ and MoS2^70^ may lead to other kinds of high-performance waveguides and devices.

## Discussion

In addition to demonstrating good optical performance under ideal conditions as discussed above, the proposed HDLNRPW also exhibits good tolerance to possible fabrication imperfections, such as the lateral misalignment between the silicon nanoridge and the upper silicon slab. Our calculations show that less than ~3.5% of the propagation distance and less than ~1.8% of the normalized optical power can be observed when the deviation of the nanoridge was varied between 0 nm and 50 nm. Meanwhile, the subwavelength mode area and high *FoM* were maintained within the considered geometric parameter range (see Supplementary Information for details). Such optical performance clearly indicates the robust modal behavior of the proposed waveguide against fabrication errors, making the HDLNRPW promising for practical applications.

**Figure 5:**
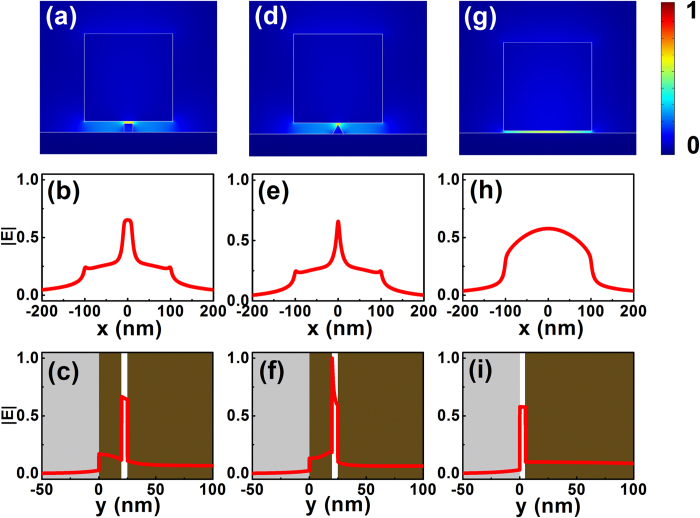
2D and 1D normalized electric field distributions for (**a**)–(**c**) HDLNRPW with a square nanoridge, (**d**)–(**f**) HDLNRPW with a triangular nanoridge, and (**g**)–(**i**) traditional HPW. For all three waveguides, the sizes of rectangular-shaped silicon slabs were *w* = *h* = 200 nm, whereas their gap sizes were 5 nm. The width of the square silicon nanoridge was 20 nm. The structure in (**d**) had an equilateral triangular silicon nanoridge of height 20 nm. For all three structures, the electric fields were normalized with respect to the power flow in each structure. The 1D field profiles show the normalized electric field along the center of the gap region.

Besides the semi-circular nanoridge-based HDLNRPW in the above case studies, our hybrid waveguide concept is readily applicable to many other structures as well. Here in Fig. 5, we show the electric field distributions of the fundamental hybrid modes guided by two different HDLNRPWs, which incorporate square and triangular-shaped silicon nanoridges, respectively. As clearly illustrated from the 2D field panels and 1D cross-sectional curves, these hybrid configurations also demonstrate stronger field enhancement inside the gap region as compared to the conventional HPW; simultaneously they feature less mode attenuation and greater figure of merit. These optical performances are similar to those of their semi-circular waveguiding counterparts.

**Figure 6:**
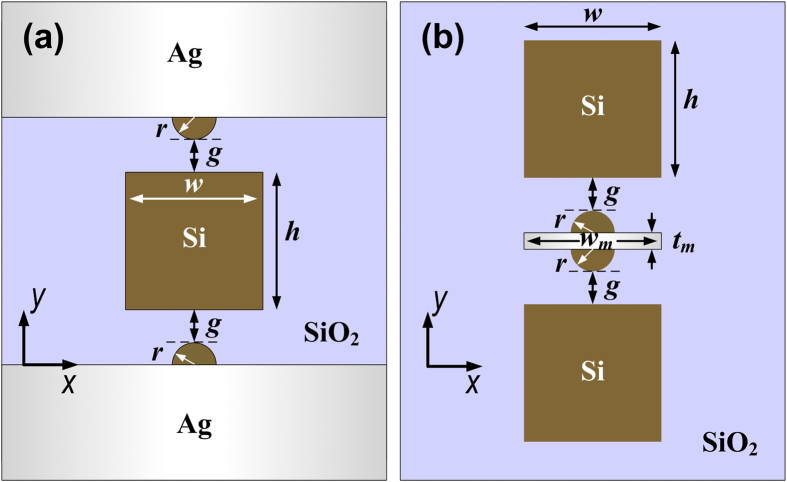
2D schematics of two typical modified HDLNRPWs. (**a**) MIM type hybrid waveguide with double silicon nanoridges; (**b**) IMI type hybrid waveguide with double silicon nanoridges.

**Figure 7:**
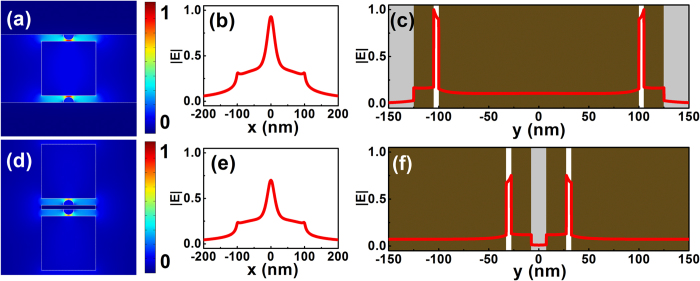
2D and 1D electric field profiles of guided low-loss plasmonic modes for MIM type and IMI type HDLNRPWs. (**a**)–(**c**) MIM type hybrid waveguide, whose geometric parameters were *w = h* = 200 nm, *r* = 20 nm, and *g* = 5 nm; (**d**)–(**f**) IMI type hybrid waveguide with structural parameters as: *w = h* = *w*_*m*_ = 200 nm, *t*_*m*_ = 15 nm, *r* = 20 nm, and *g* = 5 nm.

In Fig. 6, we plot the geometries of two other types of modified hybrid nanoridge waveguides, which were obtained by combining the HDLNRPW concept with a metal-insulator-metal (MIM) waveguide or an insulator-metal-insulator (IMI) configuration. Both configurations can be realized using modern micro-nanofabrication technologies (See Supplementary Information for details). The electric field distributions of low-loss hybrid modes guided by typical MIM-HDLNRPW and IMI-HDLNRPW are shown in Fig. 7. Significant local field enhancement was observed inside the gap regions for both waveguides. Our calculations further show that the MIM-type hybrid nanoridge structure is capable of providing ultra-tight confinement of the optical field (e.g., larger *NOP*), whereas the IMI-type hybrid configuration features extremely low propagation loss, with centimeter-range propagation distance at appropriate geometries.

In summary, we have proposed and investigated a new class of plasmonic waveguiding platform based on the combination of dielectric-loaded configurations and hybrid plasmonic structures. Through optimizations of key structural parameters, high-performance waveguiding with both large propagation length and subwavelength mode size can be achieved at a telecommunication wavelength using our proposed HDLNRPW. Compared with conventional HPWs, our proposed structure demonstrates not only lower propagation loss but also a greater figure of merit under optimal conditions. Furthermore, we show that the HDLNRPW is also highly tolerant to possible fabrication imperfections, thereby rendering it a good candidate for building practical photonic components. Finally, we discussed the possibility of extending our current waveguide concept into several other types of plasmonic configurations, providing guidelines for future studies.

## Methods

The modal characteristics of HDLNRPWs are investigated numerically by solving the Helmholtz equation using the eigenmode solver of the finite element method (FEM) based software COMSOL^TM^. A scattering boundary condition was applied to mimic the open boundary. Convergence tests ensured that the numerical boundaries and meshing did not interfere with the solutions. The modal properties were characterized by a complex wave vector, whose parallel component defines the propagating constant with *β* + i*α*. Here, *β* and *α* are the phase and attenuation constants, respectively. The real part of the modal effective index was calculated by *n*_*eff*_ = Re(*N*_*eff*_) = *β*/*k*_*0*_, where *k*_*0*_ is the vacuum wavevector. The propagation length was defined by *L* = 1/2α = *λ*/[4*π*Im(*N*_*eff*_)], whereas the effective mode area was defined as the ratio of the total mode energy and the maximum electromagnetic energy density^30^:


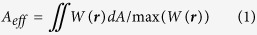


In order to accurately account for the energy in the metallic region, the electromagnetic energy density *W(**r***) is defined as^22^,^30^:


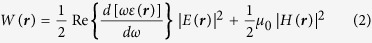


In equation (2), *E(**r***) and *H(**r***) are the electric and magnetic fields, ε(***r***) is the electric permittivity and *μ*_*0*_ is the vacuum magnetic permeability.

The normalized effective mode area is defined as *A*_*eff*_/*A*_*0*_ where *A*_*0*_ = λ^2^/4 is the diffraction-limited mode area in free space. The figure of merit was defined as the ratio of the propagation length to the diameter of the effective mode area (2(*A*_*eff*_/*π*)½). The normalized optical power was defined as the ratio of the power inside the whole gap region to the total power of the waveguide.

## Additional Information

**How to cite this article**: Zhang, B. *et al*. Hybrid Dielectric-loaded Nanoridge Plasmonic Waveguide for Low-Loss Light Transmission at the Subwavelength Scale. *Sci. Rep.* **7**, 40479; doi: 10.1038/srep40479 (2017).

**Publisher's note:** Springer Nature remains neutral with regard to jurisdictional claims in published maps and institutional affiliations.

## Supplementary Material

Supplementary Information

## References

[b1] Gramotnev D. K. & Bozhevolnyi S. I. Plasmonics beyond the diffraction limit. Nat. Photon. 4, 83–91 (2010).

[b2] Brongersma M. L. & Shalaev V. M. The Case for Plasmonics. Science 328, 440–441 (2010). 20413483 10.1126/science.1186905

[b3] Erickson D., Serey X., Chen Y.-F. & Mandal S. Nanomanipulation using near field photonics. Lab Chip 11, 995–1009 (2011). 21243158 10.1039/c0lc00482k

[b4] Zhao C. L., Liu Y. M., Zhao Y. H., Fang N. & Huang T. J. A reconfigurable plasmofluidic lens. Nat. Commun. 4 (2013). 10.1038/ncomms3305 PMC3998757 23929463

[b5] Xu X. . Near-field enhanced plasmonic-magnetic bifunctional nanotubes for single cell bioanalysis. Adv. Funct. Mater. 23, 4332–4338 (2013).

[b6] O’Dell D., Serey X. & Erickson D. Self-assembled photonic-plasmonic nanotweezers for directed self-assembly of hybrid nanostructures. Appl. Phys. Lett. 104, 043112 (2014).

[b7] Wang M. . Plasmofluidics: Merging Light and Fluids at the Micro-/Nanoscale. Small 11, 4423–4444 (2015). 26140612 10.1002/smll.201500970 PMC4856436

[b8] Han Z. H. & Bozhevolnyi S. I. Radiation guiding with surface plasmon polaritons. Rep. Prog. Phys. 76, 016402 (2013). 23249644 10.1088/0034-4885/76/1/016402

[b9] Maier S. A. . Local detection of electromagnetic energy transport below the diffraction limit in metal nanoparticle plasmon waveguides. Nat. Mater. 2, 229–232 (2003). 12690394 10.1038/nmat852

[b10] Ditlbacher H. . Silver nanowires as surface plasmon resonators. Phys. Rev. Lett. 95, 257403 (2005). 16384506 10.1103/PhysRevLett.95.257403

[b11] Zhang S. P. . Chiral Surface Plasmon Polaritons on Metallic Nanowires. Phys. Rev. Lett. 107, 096801 (2011). 21929259 10.1103/PhysRevLett.107.096801

[b12] Wei H. & Xu H. X. Nanowire-based plasmonic waveguides and devices for integrated nanophotonic circuits. Nanophotonics 1, 155–169 (2012).

[b13] Wang Y. P., Ma Y. G., Guo X. & Tong L. M. Single-mode plasmonic waveguiding properties of metal nanowires with dielectric substrates. Opt. Express 20, 19006–19015 (2012). 23038541 10.1364/OE.20.019006

[b14] Bian Y. S. & Gong Q. H. Metallic Nanowire-Loaded Plasmonic Slot Waveguide for Highly Confined Light Transport at Telecom Wavelength. IEEE J. of Quantum Electron. 49, 870–876 (2013).

[b15] Berini P. Long-range surface plasmon polaritons. Adv. Opt. Photonics 1, 484–588 (2009).

[b16] Moreno E., Rodrigo S. G., Bozhevolnyi S. I., Martin-Moreno L. & Garcia-Vidal F. J. Guiding and focusing of electromagnetic fields with wedge plasmon polaritons. Phys. Rev. Lett. 100, 023901 (2008). 18232867 10.1103/PhysRevLett.100.023901

[b17] Boltasseva A. . Triangular metal wedges for subwavelength plasmon-polariton guiding at telecom wavelengths. Opt. Express 16, 5252–5260 (2008). 18542627 10.1364/oe.16.005252

[b18] Bozhevolnyi S. I., Volkov V. S., Devaux E., Laluet J. Y. & Ebbesen T. W. Channel plasmon subwavelength waveguide components including interferometers and ring resonators. Nature 440, 508–511 (2006). 16554814 10.1038/nature04594

[b19] Volkov V. S., Bozhevolnyi S. I., Devaux E., Laluet J. Y. & Ebbesen T. W. Wavelength selective nanophotonic components utilizing channel plasmon polaritons. Nano Lett. 7, 880–884 (2007). 17352507 10.1021/nl070209b

[b20] Veronis G. & Fan S. H. Guided subwavelength plasmonic mode supported by a slot in a thin metal film. Opt. Lett. 30, 3359–3361 (2005). 16389831 10.1364/ol.30.003359

[b21] Pile D. F. P. . Two-dimensionally localized modes of a nanoscale gap plasmon waveguide. Appl. Phys. Lett. 87, 261114 (2005).

[b22] Dionne J. A., Sweatlock L. A., Atwater H. A. & Polman A. Plasmon slot waveguides: Towards chip-scale propagation with subwavelength-scale localization. Phys. Rev. B 73, 035407 (2006).

[b23] Steinberger B. . Dielectric stripes on gold as surface plasmon waveguides. Appl. Phys. Lett. 88, 094104 (2006).

[b24] Holmgaard T. & Bozhevolnyi S. I. Theoretical analysis of dielectric-loaded surface plasmon-polariton waveguides. Phys. Rev. B 75, 245405 (2007).

[b25] Krasavin A. V. & Zayats A. V. Passive photonic elements based on dielectric-loaded surface plasmon polariton waveguides. Appl. Phys. Lett. 90, 211101 (2007).

[b26] Gosciniak J. . Fiber-coupled dielectric-loaded plasmonic waveguides. Opt. Express 18, 5314–5319 (2010). 20389544 10.1364/OE.18.005314

[b27] Chu H. S., Li E. P., Bai P. & Hegde R. Optical performance of single-mode hybrid dielectric-loaded plasmonic waveguide-based components. Appl. Phys. Lett. 96, 221103 (2010).

[b28] Kumar A. . Dielectric-loaded plasmonic waveguide components: Going practical. Laser Photonics Rev. 7, 938–951 (2013).

[b29] Zia R., Selker M. D., Catrysse P. B. & Brongersma M. L. Geometries and materials for subwavelength surface plasmon modes. J. Opt. Soc. Am. A 21, 2442–2446 (2004). 10.1364/josaa.21.002442 15603083

[b30] Oulton R. F., Sorger V. J., Genov D. A., Pile D. F. P. & Zhang X. A hybrid plasmonic waveguide for subwavelength confinement and long-range propagation. Nat. Photon. 2, 496–500 (2008).

[b31] Sorger V. J. . Experimental demonstration of low-loss optical waveguiding at deep sub-wavelength scales. Nat. Commun. 2, 331 (2011).

[b32] Alam M. Z., Aitchison J. S. & Mojahedi M. A marriage of convenience: Hybridization of surface plasmon and dielectric waveguide modes. Laser Photonics Rev. 8, 394–408 (2014).

[b33] Oulton R. F. . Plasmon lasers at deep subwavelength scale. Nature 461, 629–632 (2009). 19718019 10.1038/nature08364

[b34] Wu M., Han Z. H. & Van V. Conductor-gap-silicon plasmonic waveguides and passive components at subwavelength scale. Opt. Express 18, 11728–11736 (2010). 20589033 10.1364/OE.18.011728

[b35] Li Q., Song Y., Zhou G., Su Y. K. & Qiu M. Asymmetric plasmonic-dielectric coupler with short coupling length, high extinction ratio, and low insertion loss. Opt. Lett. 35, 3153–3155 (2010). 20890317 10.1364/OL.35.003153

[b36] Ma R.-M., Oulton R. F., Sorger V. J., Bartal G. & Zhang X. Room-temperature sub-diffraction-limited plasmon laser by total internal reflection. Nat. Mater. 10, 110–113 (2011). 21170028 10.1038/nmat2919

[b37] Ma R.-M., Yin X., Oulton R. F., Sorger V. J. & Zhang X. Multiplexed and electrically modulated plasmon laser circuit. Nano Lett. 12, 5396–5402 (2012). 22989288 10.1021/nl302809a

[b38] Zhu S. Y., Lo G. Q. & Kwong D. L. Components for silicon plasmonic nanocircuits based on horizontal Cu-SiO2-Si-SiO2-Cu nanoplasmonic waveguides. Opt. Express 20, 5867–5881 (2012). 22418464 10.1364/OE.20.005867

[b39] Lou F., Wang Z. C., Dai D. X., Thylen L. & Wosinski L. Experimental demonstration of ultra-compact directional couplers based on silicon hybrid plasmonic waveguides. Appl. Phys. Lett. 100, 241105 (2012).

[b40] Hou Y., Renwick P., Liu B., Bai J. & Wang T. Room temperature plasmonic lasing in a continuous wave operation mode from an InGaN/GaN single nanorod with a low threshold. Sci. Rep. 4, 5014 (2014). 24852881 10.1038/srep05014 PMC4031474

[b41] Sorger V. J. . Strongly Enhanced Molecular Fluorescence inside a Nanoscale Waveguide Gap. Nano Lett. 11, 4907–4911 (2011). 21978206 10.1021/nl202825s

[b42] Yang X. D., Liu Y. M., Oulton R. F., Yin X. B. & Zhang X. A. Optical forces in hybrid plasmonic waveguides. Nano Lett. 11, 321–328 (2011). 21229998 10.1021/nl103070n

[b43] Zhang J., Zhao P., Cassan E. & Zhang X. Phase regeneration of phase-shift keying signals in highly nonlinear hybrid plasmonic waveguides. Opt. Lett. 38, 848–850 (2013). 23503236 10.1364/OL.38.000848

[b44] Li H., W. Noh J., Chen Y. & Li M. Enhanced optical forces in integrated hybrid plasmonic waveguides. Opt. Express 21, 11839–11851 (2013). 23736405 10.1364/OE.21.011839

[b45] Diaz F. . Sensitive method for measuring third order nonlinearities in compact dielectric and hybrid plasmonic waveguides. Opt. Express 24, 545–554 (2016). 26832285 10.1364/OE.24.000545

[b46] Dai D. X. & He S. L. A silicon-based hybrid plasmonic waveguide with a metal cap for a nano-scale light confinement. Opt. Express 17, 16646–16653 (2009). 19770880 10.1364/OE.17.016646

[b47] Bian Y. S., Zheng Z., Zhao X., Zhu J. S. & Zhou T. Symmetric hybrid surface plasmon polariton waveguides for 3D photonic integration. Opt. Express 17, 21320–21325 (2009). 19997371 10.1364/OE.17.021320

[b48] Kim J. T. CMOS-Compatible Hybrid Plasmonic Waveguide for Subwavelength Light Confinement and On-Chip Integration. IEEE Photon. Technol. Lett. 23, 206–208 (2011).

[b49] Kwon M. S. Metal-insulator-silicon-insulator-metal waveguides compatible with standard CMOS technology. Opt. Express 19, 8379–8393 (2011). 21643089 10.1364/OE.19.008379

[b50] Chen L. . A Silicon-Based 3-D Hybrid Long-Range Plasmonic Waveguide for Nanophotonic Integration. J. Lightwave Technol. 30, 163–168 (2012).

[b51] Bian Y. S. . Highly Confined Hybrid Plasmonic Modes Guided by Nanowire-embedded-metal Grooves for Low-loss Propagation at 1550nm. IEEE J. Select. Topics Quantum Electron. 19, 4800106 (2013).

[b52] Huang C. C. Metal Nanoridges in Hollow Si-Loaded Plasmonic Waveguides for Optimal Mode Properties and Ultra-Compact Photonic Devices. IEEE J. Select. Topics Quantum Electron. 20, 4600409 (2014).

[b53] Bian Y. S. & Gong Q. H. Deep-subwavelength light confinement and transport in hybrid dielectric-loaded metal wedges. Laser Photonics Rev. 8, 549–561 (2014).

[b54] Liang H. W., Ruan S. C., Zhang M., Su H. & Li I. L. Modified surface plasmon polaritons for the nanoconcentration and long-range propagation of optical energy. Sci. Rep. 4 (2014).

[b55] Gui C. C. & Wang J. Wedge hybrid plasmonic THz waveguide with long propagation length and ultra-small deep-subwavelength mode area. Sci. Rep. 5 (2015). 10.1038/srep11457 PMC4496726 26155782

[b56] Bian Y. & Gong Q. Metallic-nanowire-loaded silicon-on-insulator structures: a route to low-loss plasmon waveguiding on the nanoscale. Nanoscale 7, 4415–4422 (2015). 25648863 10.1039/c4nr06890d

[b57] Ma Y. Q., Farrell G., Semenova Y. & Wu Q. A Hybrid Wedge-To-Wedge Plasmonic Waveguide With Low Loss Propagation and Ultra-Deep-Nanoscale Mode Confinement. Journal of Lightwave Technology 33, 3827–3835 (2015).

[b58] Huang Q., Bao F. & He S. Nonlocal effects in a hybrid plasmonic waveguide for nanoscale confinement. Opt. Express 21, 1430–1439 (2013). 23389124 10.1364/OE.21.001430

[b59] Bian Y. S., Zheng Z., Zhao Y. S., Wang G. J. & Li S. N. Nanoscale light guiding in a silicon-based hybrid plasmonic waveguide that incorporates an inverse metal ridge. Phys. Status Solidi a 210, 1424–1428 (2013).

[b60] Ma R. M., Oulton R. F., Sorger V. J. & Zhang X. Plasmon lasers: coherent light source at molecular scales. Laser Photonics Rev. 7, 1–21 (2013).

[b61] Eftekhari F. . Nanoholes as nanochannels: flow-through plasmonic sensing. Anal. Chem. 81, 4308–4311 (2009). 19408948 10.1021/ac900221y

[b62] Measor P. . Multi-mode mitigation in an optofluidic chip for particle manipulation and sensing. Opt. Express 17, 24342–24348 (2009). 20052144 10.1364/OE.17.024342 PMC2860178

[b63] Schmidt H. Liquid-Core Waveguide Sensors. in Optical Guided-Wave Chemical and Biosensors Ii Vol. 8 195–219 (2010).

[b64] Zhang X. . Coupled optofluidic ring laser for ultrahigh-sensitive sensing. Opt. Express 19, 22242–22247 (2011). 22109066 10.1364/OE.19.022242

[b65] Ren L. . Ultrasensitive label-free coupled optofluidic ring laser sensor. Opt. Lett. 37, 3873–3875 (2012). 23041888 10.1364/ol.37.003873

[b66] Escobedo C., Brolo A. G., Gordon R. & Sinton D. Optofluidic concentration: plasmonic nanostructure as concentrator and sensor. Nano Lett. 12, 1592–1596 (2012). 22352888 10.1021/nl204504s

[b67] Xu X., Kim K., Liu C. & Fan D. Fabrication and Robotization of Ultrasensitive Plasmonic Nanosensors for Molecule Detection with Raman Scattering. Sensors 15, 10422–10451 (2015). 25946633 10.3390/s150510422 PMC4481927

[b68] Liu B., Liu Y. & Shen S. Thermal plasmonic interconnects in graphene. Phys. Rev. B 90, 195411 (2014).

[b69] Li Z. . Graphene plasmonic metasurfaces to steer infrared light. Sci. Rep. 5 (2015). 10.1038/srep12423 PMC5378890 26201677

[b70] Yin X. . Edge nonlinear optics on a MoS2 atomic monolayer. Science 344, 488–490 (2014). 24786072 10.1126/science.1250564

