# Supplementary material for: Hybrid Dielectric-loaded Nanoridge Plasmonic Waveguide for Low-Loss Light Transmission at the Subwavelength Scale
Source: Sci Rep. 2017 Jan 16;7:40479. doi: 10.1038/srep40479 (PMC5238436; doi:10.1038/srep40479)
Supplement: Supplementary Information [file srep40479-s1.pdf]

Supplementary Information for:

Hybrid Dielectric-loaded Nanoridge Plasmonic Waveguide for Low-Loss Light Transmission at the Subwavelength Scale

Bin Zhang[a,b,*], Yusheng Bian[a,*], Liqiang Ren[a], Feng Guo[a], Shiyang Tang[a], Zhangming Mao[a], Xiaomin Liu[b], Jinju Sun[b], Jianying Gong[a,c], Xiasheng Guo[a,d], and Tony Jun Huang[a,e1]

[a] Department of Engineering Science and Mechanics, The Pennsylvania State University, University Park, PA 16802, USA

[b] Department of Fluid Machinery and Engineering, School of Energy and Power Engineering, Xi'an Jiaotong University, Xi'an 710049, P. R. China

[c] MOE Key Laboratory of Thermo-Fluid Science and Engineering, School of Energy and Power Engineering, Xi'an Jiaotong University, Xi'an 710049, P. R. China

[d] Key Laboratory of Modern Acoustics (MOE), Department of Physics, Nanjing University, Nanjing 210093, P.R. China

[e] Department of Mechanical Engineering and Materials Science, Duke University, Durham, NC 27708, USA.

[*] The authors contributed equally to this work.

[1] Corresponding Author. Email: tony.huang@duke.edu

**HDLNRPW**	g = 2 nm	g = 5 nm	g = 10 nm	g = 25 nm	g = 40 nm	g = 50 nm	g = 80 nm	g = 100 nm
L (μm)	41	46	54	74	93	105	139	161
FoM	645	567	527	567	673	735	887	965
**HPW**	g = 2 nm	g = 5 nm	g = 10 nm	g = 25 nm	g = 40 nm	g = 50 nm	g = 80 nm	g = 100 nm
L (μm)	21	27	35	57	76	89	125	149
FoM	233	242	315	497	620	690	851	937

Supplementary Table 1: Performance comparison between hybrid dielectric-loaded nanoridge plasmonic waveguide (HDLNRPW) and traditional hybrid plasmonic waveguide (HPW). The results are based on the curves in Fig. 3, where the largest L and FoM that could be achieved within the range of the considered dimensions are listed for both waveguides.

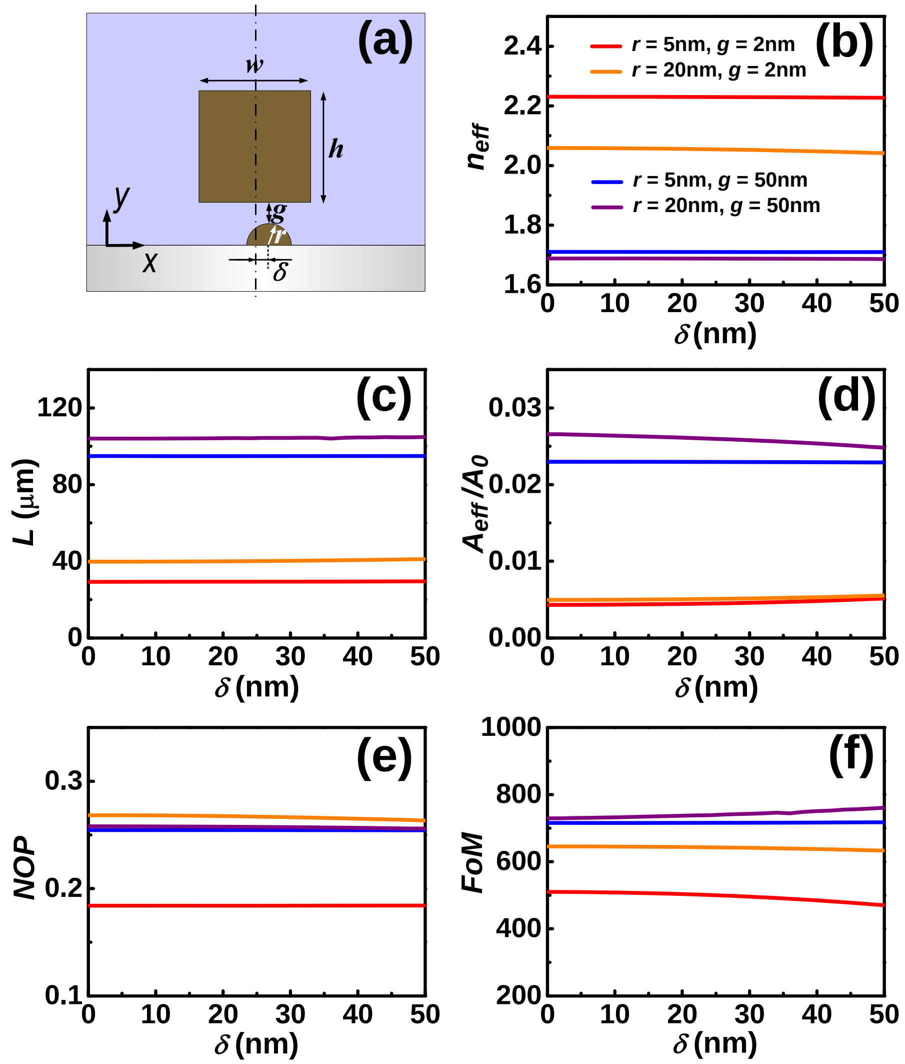

Supplementary Figure 1: Modal properties of hybrid dielectric-loaded nanoridge waveguide with a laterally displaced silicon nanoridge: (a) 2D schematic of the studied waveguide configuration with a deviated silicon nanoridge, where the displacement of the nanoridge is denoted as δ; (b) modal effective index (n_{eff}); (c) propagation length (L); (d) normalized mode area (A_{eff}/A_0); (d) normalized optical power (NOP) inside the gap region; (f) figure of merit (FoM).

The fabrication processes of the insulator-metal-insulator type HDLNRPW are shown schematically in Suppl. Fig.1, which include the following steps: (a) deposition of a PMMA layer on a silica substrate; (b) definition of a finite silver stripe using E-beam lithography, metal evaporation and lift off process; (c) fabrication of a semi-circular silicon nanowire on the silver stripe, which may be accomplished via laser induced chemical vapor deposition [1]; (d) deposition of the silica gap layer, formation of the silicon nanoridge (defined by lithography) and deposition of the silica cladding; (e) dissolving the PMMA layer in acetone; (f) repeating the steps in (c)-(d) to form the entire structure.

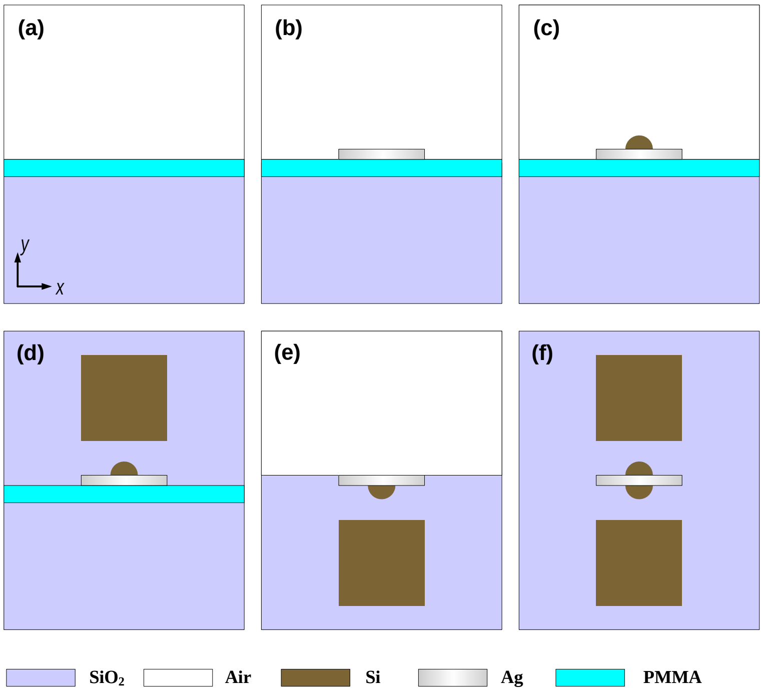

Supplementary Figure 2: Fabrication processes of the insulator-metal-insulator type HDLNRPW

The fabrication processes of the metal-insulator-metal type HDLNRPW are shown schematically in Suppl. Fig.2, including: (a) fabrication of a semi-circular silicon nanowire on a silver substrate using laser induced chemical vapor deposition; (b) deposition of the lower silica gap layer and formation of the silicon nanoridge (defined by lithography); (c) deposition of the silica cladding and the upper gap layer; (d) formation of the upper semi-circular silicon nanowire; (e) fabrication of the thin

silica layer; (f) deposition of the silver cladding.

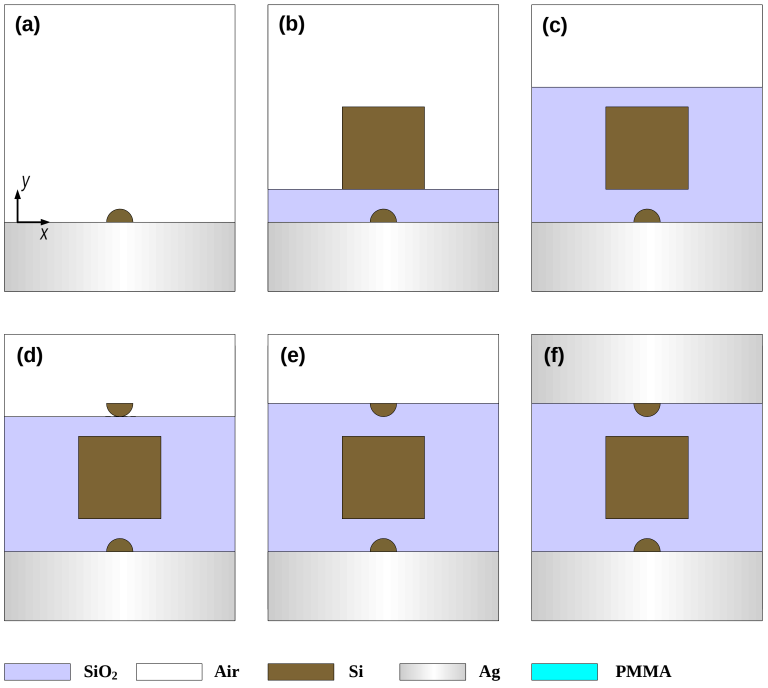

Supplementary Figure 3: Fabrication processes of the metal-insulator-metal type HDLNRPW

References

1. J. I. Mitchell, N. Zhou, W. Nam, L. M. Traverso, and X. F. Xu, "Sub-diffraction Laser Synthesis of Silicon Nanowires," Scientific Reports **4** (2014).
